# Antityrosinase Activity of *Combretum micranthum*, *Euphorbia hirta* and *Anacardium occidentale* Plants: Ultrasound Assisted Extraction Optimization and Profiling of Associated Predominant Metabolites

**DOI:** 10.3390/molecules25112684

**Published:** 2020-06-09

**Authors:** Hussein Zeitoun, Zareen Khan, Kaushik Banerjee, Dominique Salameh, Roger Lteif

**Affiliations:** 1 Unité de Technologie et Valorisation Alimentaire, Centre d’Analyses et de Recherche, Université Saint-Joseph, Faculté des sciences, Campus des Sciences et Technologies, Mar Roukos, Mkallès, P.O. Box 11- 514, Riad El Solh, Beirut 1107 2050, Lebanon; Dominique.salameh@usj.edu.lb (D.S.); Roger.lteif@usj.edu.lb (R.L.); 2 National Reference Laboratory, ICAR-National Research Centre for Grapes, Pune 412307, India; Zareenk19@gmail.com (Z.K.); kbgrape@yahoo.com (K.B.)

**Keywords:** *Combretum micranthum*, *Euphorbia hirta*, *Anacardium occidentale*, ultrasound assisted-extraction, metabolic profiling, antityrosinase activity

## Abstract

Tyrosinase is an important component of the enzyme polyphenol oxidase, which upon contact with the phenolic substrates forms the pigment melanin and induces undesirable food browning. The phenolic and triterpenoid compounds that naturally occur in plants are well known as tyrosinase inhibitors. *Combretum micranthum* (CM) leaves, *Euphorbia hirta* (EH) plant, and *Anacardium occidentale* (AO) fruits are traditionally known to have potential anti-tyrosinase activities. The aim of this study was to optimize the ultrasound-assisted extraction of secondary metabolites from these matrices, and to evaluate in tubo the antityrosinase activity of these extracts. Efforts were also taken to profile the secondary metabolites, mainly the phenolic and triterpenoid compounds, in order to understand their probable association with tyrosinase inhibition. The optimal ultrasound-assisted extraction conditions for simultaneous extraction of phenolic, and triterpenoid compounds were determined. The aqueous fraction of these extracts showed significant antityrosinase activity, with the CM leaves exhibiting the strongest inhibitory effect (IC_50_ of 0.58 g·L^−1^). The predominant metabolic compounds from these natural extracts were putatively identified by using a high-resolution quadrupole-time of flight (QToF) LC-MS instrument. The high-resolution accurate mass-based screening resulted in identification of 88 predominant metabolites, which included dihydrodaidzein-7-*O*-glucuronide, micromeric acid, syringic acid, morin, quercetin-3-*O*-(6″-malonyl-glucoside), 4-hydroxycoumarin, dihydrocaffeic acid-3-*O*-glucuronide, to name some, with less than 5 ppm of mass error.

## 1. Introduction

The browning effect can degrade the appearance, nutritional value, shelf life and marketability of fruits and vegetables [1]. In the food industry the use of tyrosinase enzyme inhibitors as anti-browning agents is not common due to the concerns related to their food safety, off-flavors, and lack of economic feasibility. The most used anti-browning compounds include ascorbic acid, sodium chloride, L-cysteine, and sodium metabisulfite. Although the sulfite-containing compounds are also known for their anti-browning effects, their use might cause allergic reactions to consumers, and therefore, have been banned by the U.S. Food and Drug Administration since 1986. The high importance of food safety in the industry leads researchers across the world to a quest for natural plant-based browning inhibitors as they are anticipated to be free of unkind side effects [2,3].

The anti-browning agents that are commonly used in food industry include butylated hydroxytoluene, butylated hydroxyanisole, and propyl gallate, however, these compounds are suspected to cause liver damage, and carcinogenesis [4,5]. Therefore, it became necessary to find alternatives to these chemical agents. In literature, the secondary metabolites in plant extracts have been reported to provide a safer alternative to food industries in preventing browning of foods and beverages [4]. It has been demonstrated that the tyrosinase inhibitors in plant extracts work synergistically, and provide browning inhibitory activities [6]. Since plant extracts contain numerous phenolic and triterpenoid compounds with potential antityrosinase activities, they are expected to provide a high inhibitory effect on browning reactions. Lim et al. examined the inhibitory effects of chemical agents vis-à-vis natural products on the polyphenol oxidase activity in sweet potatoes, and reported a slightly lower inhibition in comparison to the chemical agents [3].

The distribution of phenolic compounds in the plant kingdom is widespread and includes a wide range of molecules with diverse chemical structures and functions. These molecules have in their structure at least one aromatic ring grafted with one or more hydroxyl groups, which allow them to act as inhibitors of melanogenesis that prohibits the expression of tyrosinase enzyme [7,8]. A recent review on phenolic and terpenoid compounds has also shown the importance of these compounds as food additives [9]. In fact, these compounds may be regarded as food preservatives since they have antimicrobial, antioxidant, and anti-browning properties [10]. In addition, extracts containing phenolic and terpenoid compounds may be useful for the development of products with enhanced nutritional value, potential health benefits, longer shelf-life, and good sensory profile [11]. The triterpene group of compounds (include triterpenes and sterols) might accumulate in plants as glycosides (saponins) in extensive amounts. The terpenoid compounds have diverse industrial applications because of their wide variety, which range from simple (flavor and fragrance) to complex (tetraterpene and polyterpene) compounds [12]. Triterpenoids are also reported to inhibit the production of melanin because of their antityrosinase properties [8,13]. During food processing, wounding stimulates oxidation of phenolic compounds and enzymatic activity, which might lead to change in color attributes (browning) that damages the appearance of foods. This reaction is mediated by the activity of polyphenol oxidase, which upon contact with the phenolic substrates, form melanin pigment [14]. Since phenolic and triterpenoid compounds inhibit the enzymatic activity of tyrosinase (an important class of polyphenol oxidase), these plant metabolites may be useful for preventing enzymatic browning in fruits and vegetables.

Generally, chemical agents are used for the prevention of browning in plant-derived foods. Plant extracts (and/or compounds from natural sources) that have health benefits for consumers, if provide safe and effective control of browning in food products, are much appreciated. So, plant extracts have recently become a subject of high interest for their antityrosinase activity because of their richness in bioactive compounds. Researchers around the world take attempts to identify such inhibitors from plant sources considering their less toxicity and better bioavailability for food applications [6,14]. Most researchers focus on identifying plant extracts with antityrosinase activity without understanding which compounds are associated to this effect [15,16]. However, only few papers report their chemical composition [16]. It is well known that phenolic and triterpenoid compounds are the most bioactive compounds that are able to inhibit the tyrosinase enzymatic activity [6].

In this study, three plant materials from Senegal were selected as raw materials based on their bioavailability, popularity, and low cost. These include *Combretum micranthum* leaves, *Euphorbia hirta* plant and *Anacardium occidentale* fruits. The leaf of *Combretum micranthum* (CM) is widely known for its medicinal properties in traditional African medicine. However, the metabolite profile of the leaves of this plant has largely remained under-explored [17,18]. *Euphorbia hirta* (EH) is another plant, the extract of which is known for the treatment of gastrointestinal diseases, and disorders [19]. It is also used as an antidote and pain reliever for scorpion stings or snakebites [20]. However, information on the compounds that might be responsible for such bio-efficacies is scarce. Similarly, fruits of *Anacardium occidentale* (AO) [21] are becoming more and more popular as new evidences on the biological properties of its extract are being reported that include antimicrobial, anti-mutagenic, and anti-inflammatory activities. It also serves as a urease inhibitor, and exerts lipoxygenasic activity, to name some. The major classes of bioactive compounds in this fruit that have been reported so far include carotenoids, vitamin C, and polyphenols [22]. In the literature, only few investigations have been reported so far on the phenolic and triterpenoid profiling of CM leaves, EH plant, and AO fruit extracts. Some studies have reported HPLC-based identification of select phenolic compounds, which includes isolation and identification of 13 phenolic compounds in CM leaves [17], 14 flavonoids in AO fruits [22], and 17 phenolic compounds in EH plant extracts [20,23]. In this study, these three extracts were screened for the predominant phenolic compounds and other phytochemicals with a non-target approach using a high-resolution quadrupole-time of flight (QToF) LC-MS. All phenolic compounds and other phytochemicals were identified based on high-resolution accurate mass analysis with the data processing through UNIFI^®^, which is a unique compound identification software solution. The aims of this study were to establish the optimal conditions of ultrasound-assisted extraction of phenolic and triterpenoid compounds from CM leaves, EH plant, and AO fruits, measure their antityrosinase activity, and establish the profile of the predominant bioactive metabolites that might be responsible for their antityrosinase activity. 

## 2. Results and Discussion

### 2.1. Fitting the Models

The complete design consisted of twenty experiments. The average values of two responses (total phenolic and total triterpenoid contents) and variances expressed by standard variation (*n* = 3) for each plant are presented in Table 1. To measure how well our model fitted to the experimental data, the parameters such as *p*-value, F-value, and coefficient of determination (R^2^) were evaluated. The ANOVA analysis evaluated the significance of the quadratic polynomial models. The R^2^ value was always between 0 and 1. The closer the R^2^ value to 1, the stronger was the model to predict the response. For a good fit of a model, an R^2^ value of at least 80% was considered [24]. From the ANOVA (Table 2), it was found that R^2^ for CM, EH and AO for the two responses were higher than 80%. The form of the models chosen to explain the relationship between the three factors (A, B and C) and the response (TPC and TTC) were well correlated and attested so that the developed models described the true behaviour of the process. The significance of each coefficient was determined using F-value and the corresponding *p*-value. Therefore, factors A, B, AA, AC and BC showed significant effects (*p* < 0.05) on the extraction recovery of total phenolic compounds for CM. Factors A, B, AA, AC, and CC also showed significant effects (*p* < 0.05) on the extraction of total triterpenoid compounds for CM. The only two significant effects for EH on the extraction of TPC were the factors A and AA, while A, B, C, and AA had significant effects on the extraction of TTC for the same plant. Statistical analysis revealed that the significant effects concerning TPC included A, B, C, AA, and CC for AO, while the significant effects concerning TTC comprised B, C, AA, AB, AC, BB, and BC. The larger the value of F and the smaller the value of *p*, the more significant was the corresponding coefficient term. At 95% confidence level, the model was significant when *p*-value was lower than 0.05 [25]. The lack-of-fit test was used to investigate the fitness of a model. A *p*-value above 0.05 indicated suitability of the model to accurately predict the variations [24]. All models showed statistically insignificant lack of fit (*p*-value greater than 0.05) except for models fitted for CM which were very close to 0.05 but not greater (0.0497 for TPC and TTC) than 0.05. In general, a model was found to be well fitted to the experimental data if it presented a significant regression, and a non-significant lack of fit [26].

Multiple regression equations were obtained in terms of coded factors, which described the effect of three independent parameters: ethanol concentration, extraction temperature, and extraction time. The equations generated allowed prediction of TPC and TTC extraction efficiency by empirical models (Equations (1) to (6)):

TPC (CM) = 44.825 + 0.752 A + 0.280 B − 0.064 C − 0.012 AA + 0.0016 AB+ 0.0175 AC + 0.0090 BB − 0.025 BC + 0.0020 CC(1)

TTC (CM) = 51.428 + 1.363 A + 0.585 B − 2.192 C − 0.015 AA + 0.0038 AB+ 0.018 AC − 0.0038 BB − 0.0053 BC + 0.017 CC(2)

TPC (EH) = −28.733 + 0.630 A + 1.364 B + 1.101 C − 0.0082 AA + 0.0028 AB − 0.0013 AC − 0.0084 BB − 0.014 BC − 0.0050 CC(3)

TTC (EH) = 22.518 − 0.181 A − 0.240 B + 0.028 C + 0.00073 AA + 0.00085 AB+ 0.00038 AC + 0.0028 BB − 0.00033 BC − 0.00011 CC(4)

TPC (AO) = 13.273 + 0.168 A − 0.173 B − 0.237 C − 0.0021 AA + 0.00022 AB+ 0.00094 AC + 0.0017 BB + 0.0014 BC + 0.0017 CC(5)

TTC (AO) = −62.084 + 0.029 A + 2.768 B + 0.515 C − 0.0047 AA + 0.0058 AB + 0.0056 AC − 0.026 BB − 0.012 BC − 0.0029 CC(6)

### 2.2. Effect of Process Variables 

**Figure 1:**
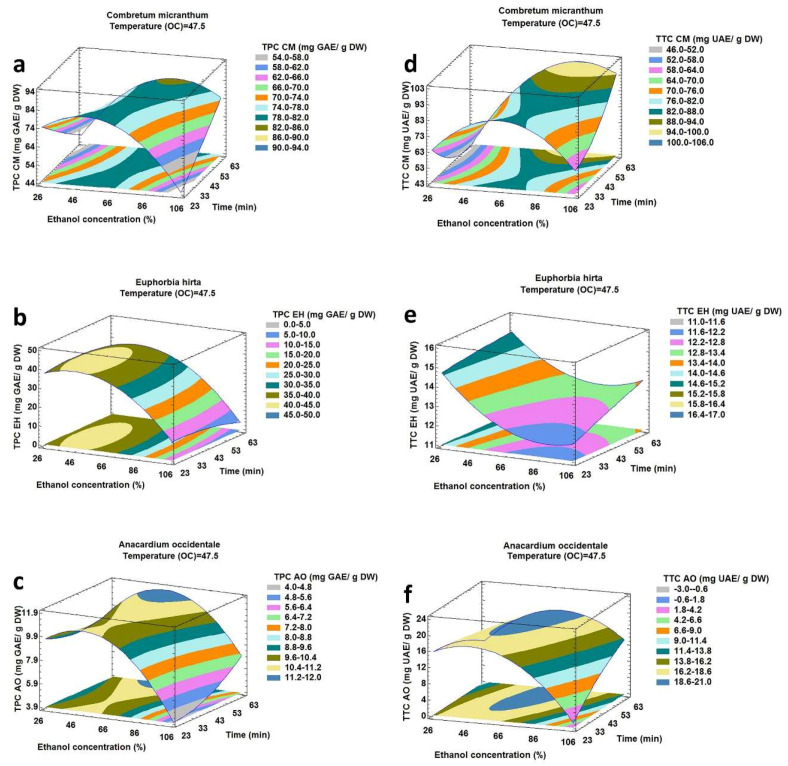
Response surface plot showing the effect of ethanol concentration and extraction time on total phenolic and total triterpenoid compounds from *Combretum micranthum* (**a**,**d**), *Euphorbia hirta* (**b**,**e**) and *Anacardium occidentale* (**c**,**f**) corresponding to extraction temperature of 47.5 °C.

**Figure 2:**
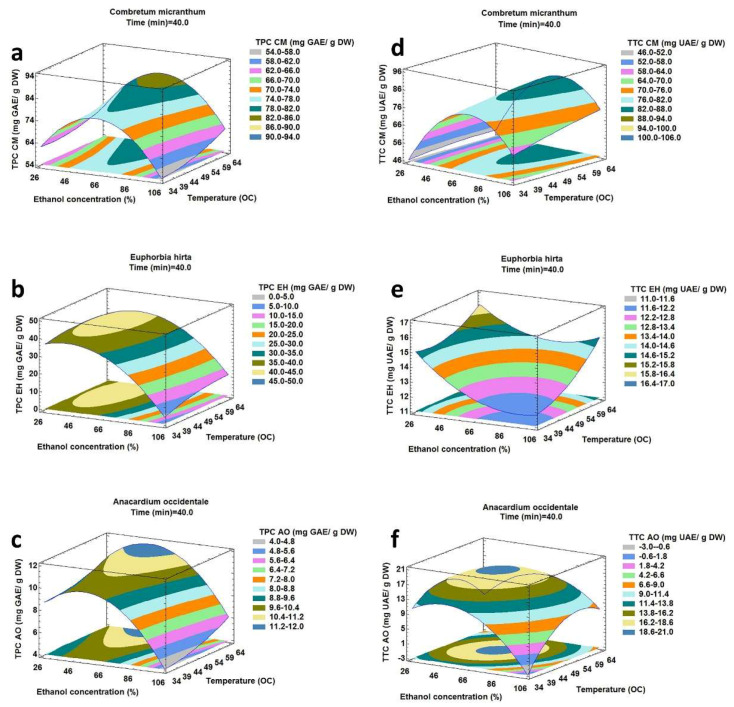
Response surface plot showing the effect of ethanol concentration and extraction temperature on total phenolic and total triterpenoid compounds from *Combretum micranthum* (**a**,**d**), *Euphorbia hirta* (**b**,**e**) and *Anacardium occidentale* (**c**,**f**) corresponding to extraction time of 40 min.

**Figure 3:**
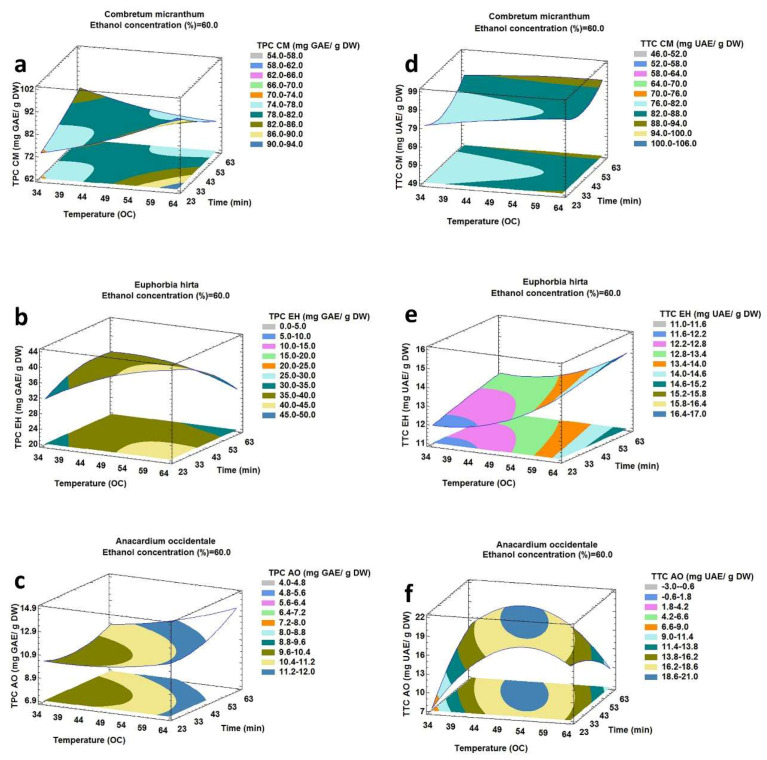
Response surface plot showing the effect of extraction temperature and extraction time on total phenolic and total triterpenoid compounds from *Combretum micranthum* (**a**,**d**), *Euphorbia hirta* (**b**,**e**) and *Anacardium occidentale* (**c**,**f**) corresponding to ethanol concentration of 60%.

The results presented in contour plots in Figure 1, Figure 2 and Figure 3 show the effect of the ultrasound-assisted extraction parameters on the responses (TPC and TTC). These graphs were drawn by maintaining one factor constant and varying the two other factors.

#### 2.2.1. Effect of Ethanol Concentration and Extraction Time on TPC and TTC

The effects of ethanol concentration (A) and extraction time (C) on TPC and TTC corresponding to the extraction temperature of 47.5 °C are reflected in Figure 1a–c, which show that TPC increased as the ethanol concentration increased.

However, beyond a certain ethanol concentration, TPC decreased significantly. In fact, extraction of phenolic compounds from plant material and their solubility depended on the nature of the solvent used and its polarity [27]. At the optimized level of ethanol concentration, TPC increased with increasing extraction time for CM and AO. A larger contact time between the solvent and the solids improved the diffusion of the compounds to be extracted [28]. For EH, TPC decreased with an increase in extraction time. This was probably due to the degradation of certain compounds after a long time of exposure to ultrasonic irradiation [29]. Figure 1d–f showed that TTC increased significantly with increasing level of ethanol concentration up to a certain value, after which it diminished progressively except for EH for which TTC always decreased with a higher ethanol concentration. The extraction yield was affected in response to variations in solvent polarity from water to ethanol. The extraction yield was also decreased with a lower water percentage due to the change in polarity and decrease in effective swelling of plant matrix [30]. At the optimum ethanol concentration, TTC always increased when ultrasonic extraction time was longer. Exposure to ultrasound irradiation for 60 min did not degrade triterpenoids, and the equilibrium of desorption was also not attainable.

#### 2.2.2. Effect of Extraction Temperature and Ethanol Concentration on TPC and TTC

The effects of temperature (B) and ethanol concentration (A) on TPC and TTC corresponding to an extraction time of 40 min are presented in Figure 2a–c.

These show that TPC increased when the extraction temperature increased. Indeed, an augmentation of extraction temperature increased the solvent diffusivity into the plant matrix and enhanced the desorption and solubility of the targeted compounds [31]. Until the optimum temperature was reached, TPC increased with increasing ethanol concentration, but beyond a certain concentration, TPC decreased significantly. These results are explained in Figure 1a–c. Figure 2d,e show that TTC always increased when the extraction temperature increased for CM and EH. Figure 2f shows that for AO, TTC increased with a rise in temperature until it reached a certain value, after which, TTC diminished progressively. These results prove that the triterpenoids content in AO degraded at a high temperature. TTC had an increasing trend with a rise in ethanol concentration. However, beyond certain level of ethanol in CM and AO, TTC started decreasing. However, the results for EH were opposite. In this case, with a rise in ethanol concentration, TTC recovery initially decreased, which subsequently increased with a further rise in ethanol concentration. For CM and AO, the results are in agreement with Figure 1d,f. For EH, the nature of triterpenoids extracted through sonication were different according to the polarity of the solvent used in the extraction. At a lower ethanol concentration, the polar triterpenoids were extracted, but at a higher ethanol concentration, less polar triterpenoids were extracted. 

#### 2.2.3. Effect of Extraction Time and Extraction Temperature on TPC and TTC

The effects of extraction temperature (B) and extraction time (C) on TPC and TTC corresponding to an ethanol concentration of 60% are presented in Figure 3a,b. 

For CM and EH, the results showed that TPC decreased when the extraction time increased at the higher temperature. On the other hand, at a lower temperature, TPC increased when extraction time increased. For AO (Figure 3c), regardless of temperature, TPC increased with increasing extraction time. In fact, ultrasound treatment disrupted the plant matrix rapidly and augmented the contact area with the solvent. Nevertheless, on a prolonged exposure to ultrasonic wave and high temperature, the targeted compounds had a chance to get oxidized or decomposed [32]. Moreover, at a lower temperature, when the vapor pressure was lower, there were few cavitation bubbles, but those collapsed with relatively high intensity and thereby enhanced the cell disruption. However, at the higher temperature, more bubbles were created, which collapsed with relatively less intensity due to a smaller pressure difference between inside and outside of the bubbles [33,34]. Figure 3e,f show that TTC increased with an increase in extraction time for EH and AO. Figure 3d showed that TTC diminished when the extraction time was increased until it reached to a minimum level. Subsequently, it increased progressively with extraction time. In addition, when the optimum extraction time was maintained, TTC always increased when extraction temperature increased. These results demonstrated the synergic effect of time and temperature in ultrasound-assisted extraction. 

### 2.3. Determination of Optimum Conditions

The optimum ultrasonic-assisted extraction conditions from CM leaves, EH plant and AO fruits were determined to maximize TPC and TTC. Based on the experimental results and the desirability function, the optimum ultrasound-assisted extraction conditions could be decided. The experiments were conducted in triplicate at the optimum conditions, and the average values were recorded. The desirability values were 0.991, 0.988 and 0.917 for CM, EH and AO, respectively. The optimal conditions were obtained with the ethanol concentrations of 65.25, 35.77 and 66.66%, at the temperatures of 60.08, 59.65, and 47.48 °C, and extraction time of 23.18, 26.12, and 56.82 min for CM, EH, and AO, respectively. At these conditions, the optimum TPC (87.04 ± 0.60; 40.43 ± 0.45 and 11.31 ± 0.28 (mg GAE/g DW)) and TTC (90.08 ± 2.57; 12.50 ± 0.58 and 18.10 ± 1.07 (mg UAE/g DW)) were obtained for CM, EH and AO, respectively. The predicted values were 88.73, 42.80, and 11.48 mg GAE/g DW for TPCs, and 89.11, 14.92, and 18.91 mg UAE/g DW for CM, EH, and AO, respectively. The experimental values were found to be in agreement with the predicted values obtained from the quadratic models developed in this study.

### 2.4. In-Tubo Tyrosinase Activity Assay 

**Figure 4:**
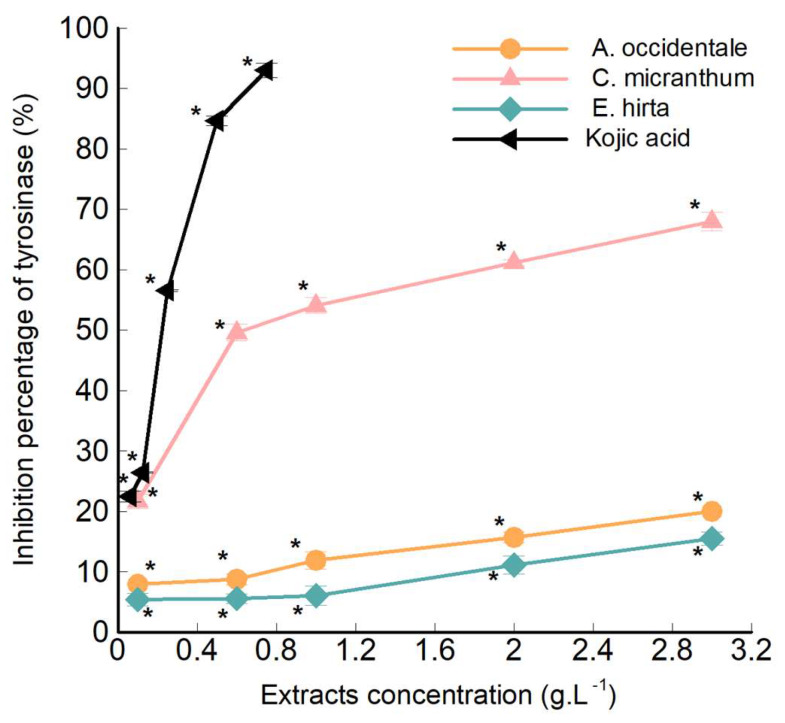
In tubo tyrosinase activity assays results for *Combretum micranthum* leaves, *Euphorbia hirta* plant and *Anacardium occidentale* fruits aqueous extract with kojic acid solution used as reference. * *p* < 0.05 indicates a significant difference between aqueous extract test and negative control.

The effects of the aqueous extracts of CM leaves, EH plant, and AO fruits on mushroom tyrosinase activity (using L-tyrosinase as a substrate), are reported in Figure 4. The in-tubo antityrosinase activity assays were carried out on the aqueous extracts considering the fact that ethanol might alter the tyrosinase activity, and the results would be inaccurate and unreproducible. Moreover, the use of a hydroethanolic extract as an anti-browning agent for food processing was not feasible since the consumption of ethanol in relatively higher level is reported to increase the risk of developing certain diseases [35]. The results show that all aqueous extracts have a significant and dose-dependent inhibitory activity of tyrosinase enzyme. The aqueous extract from CM leaves exhibited the strongest inhibitory effect. In fact, CM aqueous extract showed an IC_50_ of 0.58 g·L^−1^. However, the IC_50_ of EH and AO was not reached even at a 6-fold higher concentration. Kojic acid, the molecule used as a standard reference, showed an IC_50_ of only 0.22 g·L^−1^. In the plant extracts, the concentration of bioactive metabolites was lower than the IC_50_ value of the extracts. It is obvious since the plant extracts comprised a mixture of many compounds, whereas kojic acid is a single molecule [36]. These results were in good accordance with the previous findings and stipulated that phenolic and triterpenoid compounds could be responsible for the tyrosinase enzymatic inhibition. In fact, CM extract showed a higher concentration of both phenolic and triterpenoid compounds. However, EH extract showed a higher concentration of metabolites than AO extract but a less percentage of tyrosinase inhibition. It is not surprising as the percentage of tyrosinase inhibition of an extract is not only related to the quantity of phenolic and triterpenoid compounds, but also to its chemical composition. The influence of their chemical composition will be discussed later in this article. 

In addition to having many biological properties, these plant extracts show significant antityrosinase activity. These plants could be used as a food additive because of their safety for the consumers. Ping et al. have demonstrated that EH plant extract shows no signs of toxicity or symptoms related to oral toxicity [37]. However, it is possible that a middle-level toxicity appears at very high doses, and therefore, it is necessary to know the doses when the extract is used as an antibacterial, antioxidant or antibrowning agent in food [38]. The toxicity of AO fruits associated with dairy products has long been discussed. However, a recent study concluded that the juice and cow milk mixture are not toxic to animal cells, but the juice-yogurt mixture has some toxic effects on the liver cells [39]. CM leaf extract, which has shown the highest antityrosinase activity appeared to be the most promising one, without any symptoms of toxicity. In fact, an acute and subchronic oral toxicity assessments of CM leaves extract in Wistar rats recently showed that the oral dose up to 5000 mg/kg of CM leaves extract has no evidence of toxicity or treatment-related mortality in animals. In addition, repeated doses of the hydroalcoholic extract (1000 mg/kg) of CM leaves for 28 days showed no significant change in food and water intake. It was concluded that the risk/benefit ratio is in favour of usage of CM leaves extract [40].

### 2.5. Identification of Metabolic Compounds

A total of 88 metabolic compounds was tentatively identified in the ethanol-water extract of the plant matrices using high-resolution LC-MS (Table 3), out of which 75 were phenolic compounds (26 phenolic acids and 49 flavonoids). A total of 22, 29 and 24 phenolic compounds were detected in CM leaves, EH plant and AO fruit, respectively. All the detected compounds were tentatively identified based on the accurate mass of their precursor and one or more diagnostic product ions, each with <5 ppm of mass errors. The *m*/*z* of the observed precursor ions (from the in-house developed database of natural compounds) and their characteristic fragment ions (through in-silico fragmentation of the chemical structure of the compound, feature of UNIFI software) were matched either with the spectra of the reference standards, or with spectral library from literature (previous research articles or public databases including ChemSpider (http://www.chemspider.com), SciFinder Scholar (https://scifinder.cas.org), FooDB (http://foodb.ca/) and Phenol-Explorer (www.phenol-explorer.eu).

The identifications of all metabolic compounds were based on certain criteria as mentioned in Table 3. For instance, luteolin-7-*O*-malonylglucoside (Table 3, No. 53) was identified based on its protonated molecular ion m/z 534.10096 (mass error, 3.86 ppm with elemental composition, C_24_H_22_O_14_) and its two characteristic fragment ions (*m*/*z* 287.0559 and *m*/*z* 163.0394). Naringenin-4′-*O*-glucuronide (Table 3, No. 18 and 49) appeared at two different retention times which indicated that this compound probably appeared in two isomeric forms. Myricetin-3-*O*-glucoside (Table 3, No. 21) was identified based on its protonated molecular ion *m*/*z* 480.09039 (mass error 2.46 ppm), elemental composition (C_21_H_20_O_13_) and characteristic fragment ions with *m*/*z* 153.0183 and *m*/*z* 319.0456. In a similar manner, the other compounds (Table 3) were identified.

The results in this study were consistent when compared to previous studies. Welch et al. had also reported (−)-epigallocatechin and myricetin-3-*O*-glucoside in the leaf extract of CM [17]. According to [23,41], EH extract was earlier reported to contain chlorogenic acid and kaempferol. Besides, myricetin, (−)-epigallocatechin, myricetin-3-*O*-glucoside, and quercetin-3-*O*-galactoside were reported in previous studies in the fruit extract of AO [21,22,42]. All other compounds reported in Table 3 are reported for the first time in these plant extracts. Hence, the current results appear much more comprehensive as compared to the previous studies. There were few compounds which differed from the results of previous studies. This difference could be due to variation in methods of extraction and analysis. Moreover, the possibility of compounds being identified largely depend on the size of the database used. The UNIFI software allowed us to screen the samples against a database comprising thousands of compound entries. This offered a comprehensive screening of the compounds. Furthermore, variations in metabolite profile might also depend upon the cultivar, geographical and climatic conditions, etc. [43].

The most dominant compounds in CM leaves included dihydrodaidzein-7-*O*-glucuronide (isoflavonoid), micromeric acid (triterpenoid) and syringic acid (phenolic acid) with relative percentages of 33.38, 16.59, and 11.38%, respectively. For EH plant, the most dominant compounds included morin (flavone), quercetin-3-*O*-(6–-malonylglucoside) (flavonol), and 4-hydroxycoumarin (phenolic acid) with relative proportions of 29.38, 11.25, and 11.14%, respectively. AO fruits contained dihydrocaffeic acid-3-*O*-glucuronide (phenolic acid), micromeric acid (triterpenoid), and cucurbitacin E (triterpenoid) as the major compounds with relative percentages of 33.61, 16.05, and 7.17%, respectively. Micromeric acid was a major and common compound for both CM and AO. All three plant extracts showed to have common compounds, although in different proportional amounts. For example, naringenin 4′-*O*-glucuronide, and pelargonidin-3-*O*-coumarylglucoside were the common compounds between CM and EH. (−)-epigallocatechin and myricetin-3-*O*-glucoside were present in both CM and AO. Some of the compounds that were common between EH and AO included morin and cucurbitacin E.

A recent review article reports the tyrosinase inhibitors discovered from natural, semisynthetic, and synthetic sources in the last four decades [6]. Most of the compounds identified in this study, and many of their derivatives have been found to have great antityrosinase activity. For example, quercetin, a flavonol, is well known for its antityrosinase activity, and most of its derivatives show a similar effect [6,13]. The extracts of EH plants and AO fruits have shown significant amounts of quercetin derivatives that could be responsible for their antityrosinase activity, especially quercetin-3-*O*-(6″-malonyl-glucoside), which is the most predominant compound in EH plant extract, and have never been tested for its antityrosinase activity earlier. Quercetin-3-*O*-(6-*O*-malonyl)-β-d-glucopyranoside, and kaempferol-3-*O*-(6-*O*-malonyl)-β-d-glucopyranoside from mulberry leaves were identified as tyrosinase inhibitors [44]. The compound dihydrodaidzein-7-O-glucuronide have never been reported to have an antityrosinase activity. However, daidzein inhibited tyrosinase activity by 55.8 ± 1.4% at 100 µg·mL^−1^. We can expect to find a similar effect with daidzein derivatives, such as dihydrodaidzein-7-*O*-glucuronide, which have been found to be the most prominent compound in CM leaves [45]. Micromeric acid, an important predominant compound found in both CM leaves and AO fruits, have never been tested for its antityrosinase activity. This triterpenoid should be explored in future studies to confirm or refute its implication in the inhibition percentage of tyrosinase enzyme. Enzymatic kinetics studies have shown that morin, the most predominant compound in EH plant extract, reversibly inhibited tyrosinase in a competitive manner, and bound to tyrosinase at a single binding site by van der Waals interactions and hydrogen bonds, inducing rearrangement and conformational changes in the enzyme [46]. Asthana et al. showed that 4-hydroxycoumarin, a predominant compound in EH plant extract, was not an inhibitor of tyrosinase enzyme [47]. Dihydrocaffeic acid-3-*O*-glucuronide has never been reported to have antityrosinase activity. However, caffeic acid and its derivatives have been largely reported to have significant antityrosinase activity [48]. These results were consistent with all previous findings and allowed to explain where from the antityrosinase effect of these plant extracts came from. These findings could definitely lead to the discovery of new active compounds to inhibit the tyrosinase enzymatic activity. Now, experiments to isolate bioactive compounds responsible for the inhibition of tyrosinase enzyme are underway. These compounds could be useful in food processing industries as anti-browning agents.

## 3. Materials and Methods

### 3.1. Materials

Twenty kg of EH plant (leaves, stems, roots) were harvested from Tyre (Tyre, Lebanon) in April 2017. Twenty Kg of both CM leaves and AO fruits were harvested from Dakar (Dakar, Senegal) in July 2017. The plants were taxonomically authenticated by a botanist to confirm their genus and species. The plants were air-dried in shade for three weeks and ground until fine and homogenous particles were obtained. The same plant samples were preserved and used during the study. All chemical reagents and standards mentioned were purchased from Sigma-Aldrich (Steinheim, Germany).

### 3.2. Ultrasound-Assisted Extraction

Ultrasound-assisted extraction was performed for screening of phenolic and triterpenoid contents from leaves of CM, whole plant of EH and fruits of AO. An ultrasonic bath, Elmasonic S type S 15/H type S 15 (manufactured by Elma Hans Schmidbauer GmbH & Co. KG, Singen am Hohentwiel, Germany, bath frequency 37 KHz, power 280 W) was used. The set-up allowed regulation of temperature. The grinded plants (30, 15 and 20 g of CM, EH and AO, respectively) were placed directly into the ultrasonic bath with 300 mL of ethanol: water mixture to decide the optimal ratio utilizing the central composite design. The reaction mass was filtered under vacuum and the filtrate was collected in a volumetric flask for the determination of total phenolic and total triterpenoid contents.

### 3.3. Preliminary Study

An increase in the ratio of solvent/matrix lead to an enhancement of the gradient concentration and improved the extent of diffusion of analytes in the medium. The high gradient concentration was considered as the driving force during extraction until equilibrium was reached. Ultrasound-assisted extraction required mechanical agitation to improve mass transfer and to avoid ultrasonic intensity attenuation due to heterogeneity of the medium [49]. In order to optimize the liquid/solid ratio (mL·g^−1^) and the agitation speed (rpm or ×*g*), the maximum yield of extraction was considered. It was observed that the optimum liquid/solid ratio were 10, 20 and 15 mL·g^−1^ for CM, EH and AO, respectively. On the other hand, the optimum agitation speeds were 80, 130 and 80 rpm for CM, EH and AO, respectively.

### 3.4. Total Phenolic Content

The total phenolic content (TPC) was estimated as gallic acid equivalents (GAE), expressed as mg gallic acid equivalent/g dry weight (DW) according to the standard method [50] with slight modifications. To 0.1 mL of plant extract, 0.4 mL of distilled water and 2.5 mL of Folin-Ciocalteu solution (0.2 N) were added. After shaking, 2 mL of 7.5% (*w*/*v*) Na_2_CO_3_ was added. The solution was then incubated for 10 min at 60 °C, followed by and 10 min of incubation at −20 °C to stop the reaction. The absorbance of the samples was measured at 760 nm and compared to gallic acid calibration curve. Data are representative of three independent experiments. The linear range of gallic acid was 0.1–1 mg·mL^−1^ (R^2^ = 0.9985).

### 3.5. Total Triterpenoid Content

The total triterpenoid content (TTC) was determined by the method of Ming et al. with a slight modification and expressed as mg ursolic acid equivalent (UAE)/g dry weight (DW) [51]. The sample solution (200 µL) was heated to evaporation in a water-bath, and to it, 0.3 mL of freshly mixed 5% (*w*/*v*) vanillin-acetic solution and 1 mL sulfuric acid were added, mixed and incubated at 60 °C for 30 min. After incubation, the mixed solution was cooled and diluted to 9.3 mL with acetic acid. The absorbance was measured at 546 nm and compared to standard ursolic acid calibration curve. Data are representative of three independent experiments. The linear range of ursolic acid was 1–10 mg·mL^−1^ (R^2^ = 0.9979).

### 3.6. Antityrosinase Activity

Solution of L-tyrosine at 0.5 mg·mL^−1^, mushroom tyrosinase at 142 U·mL^−1^, as well as aqueous fraction extracts of CM leaves, EH plant and AO fruits at different concentration were prepared in phosphate buffer solution (PBS) at pH 6.6. The pure kojic acid served as the reference standard inhibitor for comparison. Briefly, 50 µL of L-tyrosine solution and 50 µL of tyrosinase solution were mixed with 50 µL of the aqueous fraction extracts or kojic acid solution. The mixture was incubated at 37 °C for 60 min, and the dopachrome was measured by UV-Vis spectroscopy at 475 nm [52]. Data are representative of three independent experiments. Data are analyzed using one-way ANOVA test. Values of * *p* < 0.05 are considered significant.

### 3.7. LC-MS [UPLC-(ESI)-QToFMS] Analysis of Metabolic Compounds

The metabolic compounds from natural extracts were identified by the method of Kumar et al. 2017 [53]. An Acquity Ultra Performance Liquid Chromatograph (UPLC) (Synapt G2 HDMS, Waters Corporations, Manchester, UK) coupled to a quadrupole time of flight mass spectrometer (QToF-MS, Synapt G2 HDMS, Waters Corporation, Manchester, UK) was used for analysis. The QToF-MS was controlled by MassLynx 4.1 software (Waters, Manchester, UK) and operated with electrospray ionization (ESI) in the positive mode at the mass resolution of 20,000, and acquisition in the MS^E^ mode provided quick switching from low energy scan at 4 V (full scan MS) to high energy scan (10–60 V ramping) during a single LC run. The low-collision energy (CE) experiments provided data about the intact molecular ion (e.g. M^+^, [M + H]^+^) and the high-CE scan generated data on the fragment ions. The source parameters included: capillary 3 kV, sampling cone 30 V, extraction cone 5 V, source temperature 120 °C, desolvation temperature 500 °C, desolvation gas flow 1000 L·h^−1^, and cone gas flow 50 L·h^−1^. Nitrogen was used both as cone gas and drying gas. The calibration of the mass spectometer was done with 0.5 mM of sodium formate. The mass correction was done using the lock spray and the reference mass leucine enkephaline (*m*/*z* 556.2771 in positive and 554.2670 in negative polarity) at 2 µg·mL^−1^ with 10 µL·min^−1^ of flow rate at an interval of 20 s. The chromatographic separation was performed on an Acquity UPLC BEH C18 column (2.1 × 100 mm, 1.8 µm, Waters Corporation, Manchester, UK) at 35 °C. The mobile phase was composed of solvent A (methanol: water (10:90, *v*/*v*) and solvent B (methanol: water (90:10, *v*/*v*) with 0.1 % formic acid in both phases. The following gradient was applied: 90% A (0–0.5 min), 50% A (0.5–4.5 min), 50–2% A (4.5–8 min), 2% A (8–11 min), 2–90% A (11–12 min), 90% A (12–15 min). The flow rate was 0.4 mL·min^−1^.

### 3.8. Data Analysis

The acquired data (*n* = 6 biological replicates) were processed in UNIFI software (version 1.7, Waters Corporation) with a screening solution workflow which helped in automated data processing to reporting the positive identifications by comparison with a database of phenolic compounds and other phytochemicals. The phenolic compounds were identified with mass errors below 5 ppm for the precursor and one or more product ion(s) having a similar mass accuracy. The product ions generated through collision induced dissociation were matched against the theoretical fragmentation pattern. Any new compounds could be added to the UNIFI software in order to create a customized library of compounds. The relative percentages of the most predominant compounds were calculated.

### 3.9. Experimental Design

The extraction of phenolic compounds and triterpenoids as a function of ethanol concentration (A), extraction temperature (B) and extraction time (C) were studied using a rotatable second order design with six replicates in the center of the experimental domain. The conditions of the independent variables studied were: A in the range of 40–80%, B in the range of 40–55 °C and C in the range 30–50 min. The two response variables to be optimized included total phenolic and total triterpenoid contents. The total number of experiments (N) in a central composite design was calculated using the following equation (Equation (7)):

(7) $$N=2^{n}+2n+x_{0}$$

Here, *n* represents the number of variables and x0 is the number of experimental central points [54]. Twenty experiments (consisting of eight factorial points, six star point and six replicates at the center) were performed to optimize the parameters. In this study, three-level-three-factor central composite design was employed, requiring 20 (*n* = 3; x_0_ = 6) experiments. A second order polynomial equation was used in order to develop an empirical model, which correlated the responses to the independent variables. The general form of second order polynomial equation (Equation (8)) was:

(8) $$R=b_{0}+\overset{n}{\underset{i=1}{\sum }}b_{i}X_{i}+\overset{n-1}{\underset{\begin{array}{c} i=1\\ j>1 \end{array}}{\sum }}\overset{n}{\underset{j=2}{\sum }}b_{ij}X_{i}X_{j}+\overset{n}{\underset{i=1}{\sum }}b_{ii}X_{i}^{2}$$

Here, R represents the predicted response, b_0_ is a constant coefficient, B_i_, b_ij_, b_ii_ are the coefficients of linear, interaction effect and squared effects respectively, *n* is the number of variables, while X_i_ and X_j_ define the independent variables [55]. Analysis of variance (ANOVA), regression analysis and response surface plots were performed in order to establish optimum conditions for total phenolic and total triterpenoid contents.

In order to reach the maximal yield, we sought to determine the optimum extraction conditions of total phenolic and total triterpenoid compounds using the desirability functions. The same weight was used for both responses (w = 1). The desirability function ranged from 0 (minimum desirability or non-desirable situations) to 1 (maximum desirability). The importance of a goal ranged between 1 to 5 (1 for the least important and 5 for the most important). In this study, all the goals were equally important and set at 3. All statistical analysis were performed using the software STATGRAPHICS^®^ Centurion XVI (Statgraphics 18, The Plains, Virginia). Triplicate experiments were carried out in the optimal condition. The experimental and predicted mean values were compared in order to determine the validity of the models.

## 4. Conclusions

This work marks the first extensive study of the most predominant phenolic compounds and other phytochemicals in the extracts of CM leaves, EH plant and AO fruits that have shown to be effective in inhibiting the enzymatic activity of tyrosinase. A total of 88 predominant metabolic compounds have been identified, including 75 polyphenols and 10 triterpenoids. On the one hand, 22, 29 and 24 polyphenols were identified in CM, EH and AO extracts, respectively. On the other hand, three, two and five triterpenoids were identified in CM, EH and AO extracts, respectively. In this paper, the extraction conditions to obtain the same plant extracts with consistent yield and quality were very well detailed. The optimal ultrasound assisted-extraction conditions recommended an ethanol concentration of 65.25, 35.77 and 66.66%, temperature of 60.08, 59.65 and 47.48 °C and extraction time of 23.18, 26.12 and 56.82 min for CM, EH and AO, respectively. All plant extracts showed significant anti-tyrosinase activity with the higher IC_50_ of 0.58 g·L^−1^ for CM extract. Moreover, the results indicated that CM extract could be considered as a good source of phenolic (87.04 ± 0.60 mg GAE/g DW) and triterpenoid (90.08 ± 2.57 mg UAE/g DW) compounds with potential utilization as bioactive ingredients in food products. Thus, researchers and industrialists who will be interested in pursuing studies on the same plant extracts will be able to do it. Recently, several benefits have been addressed to phenolic and triterpenoid compounds. The food industry that are in search of multifunctional ingredients from natural sources could be interested in using these extracts as anti-browning agents. However, we recommend evaluating the safety of these extracts by in vitro and in vivo models before their use in food products. Further studies should also focus on the isolation of these bioactive compounds and develop them as products for utilization in food industries.

## Figures and Tables

**Table 1:** Experimental data for the responses obtained from *C. micrathum* leaves, *E. hirta* plant and *A. occidentale* fruits.

Run	*Combretum micranthum* Leaves				*Euphorbia hirta* Plant				*Anacardium occidentale*			
	TPC (mg GAE/g DW)		TTC (mg UAE/g DW)		TPC (mg GAE/g DW)		TTC (mg UAE/g DW)		TPC (mg GAE/g DW)		TTC (mg UAE/g DW)	
	Average Value	Standard Deviation	Average Value	Standard Deviation	Average Value	Standard Deviation	Average Value	Standard Deviation	Average Value	Standard Deviation	Average Value	Standard Deviation
1	73.01	2.69	70.30	4.48	36.89	1.94	13.40	0.61	9.91	0.28	16.22	0.14
2	67.44	0.51	73.06	2.94	26.32	1.68	11.43	0.68	8.38	0.17	9.15	0.18
3	80.29	1.1	68.36	6.36	43.33	4.61	14.46	0.32	10.83	0.33	15.94	0.73
4	74.37	2.04	85.60	3.60	32.49	2.38	12.44	1.78	8.93	0.17	14.46	1.33
5	70.97	0.74	60.70	0.75	41.11	4.22	14.52	0.84	9.82	0.12	16.34	1.10
6	78.09	1.02	89.82	8.39	27.55	2.40	12.29	0.09	8.54	0.10	15.85	0.21
7	69.37	1.95	69.35	3.33	41.37	2.24	14.92	0.61	10.65	0.26	14.67	0.50
8	78.73	1.1	88.57	2.19	31.39	1.26	13.76	0.52	10.00	0.14	15.58	0.68
9	78.61	0.17	81.44	2.53	37.13	3.71	13.10	0.83	10.19	0.17	18.46	1.55
10	78.09	0.74	79.03	3.40	40.27	1.68	13.15	0.92	10.29	0.37	18.18	1.17
11	80.98	1.87	80.56	1.94	43.15	3.89	13.15	0.40	10.74	0.45	20.15	1.67
12	77.93	1.9	80.95	2.59	38.64	2.72	12.49	0.35	10.26	0.08	19.98	2.90
13	80.17	0.2	83.10	1.87	38.70	3.07	12.70	0.61	10.08	0.16	18.40	0.21
14	77.97	0.34	76.83	2.36	37.73	0.68	12.49	0.17	10.43	0.08	18.06	1.37
15	83.78	0.99	88.43	3.78	40.56	2.80	12.70	0.15	10.41	0.18	18.03	0.14
16	77.81	0.85	81.88	1.56	35.06	0.85	12.55	0.52	11.71	0.11	19.55	1.51
17	78.89	0.85	77.80	2.56	37.78	1.63	12.34	0.38	10.25	0.02	11.61	1.76
18	84.46	0.23	81.51	2.50	37.99	1.40	13.86	0.53	11.43	0.08	19.25	0.88
19	62.08	1.59	49.93	3.04	38.96	2.71	14.36	0.76	9.14	0.15	14.49	0.76
20	71.01	0.76	75.84	2.13	20.93	0.85	12.60	0.35	7.35	0.13	14.15	0.60

**Table 2:** Analysis of variance (ANOVA) for the second order polynomial models of *Combretum micranthum* leaves, *Euphorbia hirta* plant and *Anacardium occidentale* fruits.

	Total Phenolic Content					Total Triterpenoid Content				
Source	SS ^1^	Df ^2^	MS ^3^	F-Ratio	*p*-Value	SS	Df	MS	F-Ratio	*p*-Value
*Combretum micranthum*										
A ^4^	29.314	1	29.314	17.32	0.0088	917.124	1	917.124	196.96	<0.0001
B ^5^	37.4578	1	37.4578	22.13	0.0053	43.0225	1	43.0225	9.24	0.0288
C ^6^	4.67492	1	4.67492	2.76	0.1575	0.000796	1	0.000796	0	0.9901
AA	337.811	1	337.811	199.54	<0.0001	544.127	1	544.127	116.86	0.0001
AB	0.446512	1	0.446512	0.26	0.6294	2.62205	1	2.62205	0.56	0.4868
AC	97.7901	1	97.7901	57.76	0.0006	100.394	1	100.394	21.56	0.0056
BB	3.71458	1	3.71458	2.19	0.1986	0.66977	1	0.66977	0.14	0.7201
BC	28.7661	1	28.7661	16.99	0.0092	1.28	1	1.28	0.27	0.6225
CC	0.556849	1	0.556849	0.33	0.5911	43.0799	1	43.0799	9.25	0.0287
Lack-of-fit	42.884	5	8.57679	5.07	0.0497	117.926	5	23.5851	5.07	0.0497
Pure error	8.46488	5	1.69298			23.2815	5	4.6563		
Total (corr.)	606.903	19				1829.9	19			
R^2^	91.5392					92.2834				
R^2^ adjusted	83.9245					85.3384				
***Euphorbia hirta***										
A	414.882	1	414.882	87.43	0.0002	7.82864	1	7.82864	74.68	0.0003
B	21.3192	1	21.3192	4.49	0.0876	3.09019	1	3.09019	29.48	0.0029
C	3.44571	1	3.44571	0.73	0.4331	0.900954	1	0.900954	8.59	0.0326
AA	154.863	1	154.863	32.63	0.0023	1.22537	1	1.22537	11.69	0.0189
AB	1.36951	1	1.36951	0.29	0.6142	0.13005	1	0.13005	1.24	0.316
AC	0.567113	1	0.567113	0.12	0.7436	0.045	1	0.045	0.43	0.5413
BB	3.19556	1	3.19556	0.67	0.4492	0.356344	1	0.356344	3.4	0.1245
BC	9.05251	1	9.05251	1.91	0.2258	0.005	1	0.005	0.05	0.8358
CC	3.56558	1	3.56558	0.75	0.4257	0.00164	1	0.00164	0.02	0.9051
Lack-of-fit	28.0986	5	5.61971	1.18	0.4287	1.89648	5	0.379295	3.62	0.0922
Pure error	23.7274	5	4.74548			0.524133	5	0.104827		
Total (corr.)	658.003	19				15.9239	19			
R^2^	92.1237					84.7989				
R^2^ adjusted	85.0351					71.1179				
***Anacardium occidentale***										
A	5.1303	1	5.1303	96.19	0.0002	5.54457	1	5.54457	6.31	0.0537
B	2.41633	1	2.41633	45.3	0.0011	18.6022	1	18.6022	21.18	0.0058
C	0.724866	1	0.724866	13.59	0.0142	6.23314	1	6.23314	7.1	0.0447
AA	9.73445	1	9.73445	182.51	<0.0001	50.4501	1	50.4501	57.44	0.0006
AB	0.00845	1	0.00845	0.16	0.707	6.10751	1	6.10751	6.95	0.0461
AC	0.28125	1	0.28125	5.27	0.0701	10.0576	1	10.0576	11.45	0.0196
BB	0.131704	1	0.131704	2.47	0.1769	31.5062	1	31.5062	35.87	0.0019
BC	0.08405	1	0.08405	1.58	0.2648	6.07261	1	6.07261	6.91	0.0466
CC	0.433212	1	0.433212	8.12	0.0358	1.21737	1	1.21737	1.39	0.2921
Lack-of-fit	0.977908	5	0.195582	3.67	0.0901	22.1435	5	4.4287	5.04	0.0502
Pure error	0.266683	5	0.0533367			4.39168	5	0.878337		
Total (corr.)	20.9194	19				153.931	19			
R^2^	94.0505					82.7616				
R^2^ adjusted	88.696					67.2471				

^1^ Sum of squares; ^2^ Degrees of freedom; ^3^ Mean square; ^4^ Ethanol concentration; ^5^ Temperature; ^6^ Processing time

**Table 3:** Identification of phenolic compounds and other phytochemicals by high-resolution LC-MS (R. T.: Retention time; R. P.: Relative percentage).

No.	Compound Name	Formula	R. T. (min)	Expected Mass (*m*/*z*)	Adducts	Observed Mass (*m*/*z*)	Mass Error (ppm)	Fragments (relative%)	Detector Counts	R. P. (%)
	*C. micranthum* leaves extract									
	Phenolic acid									
1.	Syringic acid	C_9_H_10_O_5_	0.59	198.05282	−e	198.05279	2.60	182.0572 (100%)	133170.27	11.38
2.	*p*-Coumaric acid ethyl ester	C_11_H_12_O_3_	1.56	192.07864	+H	193.08632	2.06	175.0755 (100%)	35086.45	3.00
3.	Sesamol	C_7_H_6_O_3_	2.02	138.03169	+H	139.03933	2.61	121.0287 (100%)	24158.38	2.06
4.	Dihydrocaffeic acid-3-*O*-glucuronide	C_15_H_18_O_10_	0.62	358.09000	+Na	381.07991	1.81	198.0526 (100%)	19538.90	1.67
5.	Prodelphinidin trimer GC-C-C	C_45_H_38_O_20_	2.28	898.19564	+H, +Na	899.20573	3.13	729.1436 (100%), 605.1285 (80%)	14474.68	1.24
6.	*p*-Coumaric acid	C_9_H_8_O_3_	3.01	164.04734	+H	165.05504	2.56	147.0443 (100%), 119.0493 (60%)	9977.19	0.85
7.	Vanillic acid	C_8_H_8_O_4_	2.03	168.04226	+H	169.04982	1.69	139.0393 (100%), 151.0393 (80%	3730.41	0.32
8.	Eugenol 1	C_10_H_12_O_2_	1.56	164.08373	+H	165.09132	1.92	147.0807 (100%),	2514.33	0.21
	Isoflavonoid									
9.	Dihydrodaidzein-7-*O*-glucuronide	C_21_H_20_O_10_	4.70	432.10565	+H, +Na	433.11431	3.20	415.1037 (60%), 313.0714 (100%), 283.0608 (100%)	390762.65	33.38
	Anthocyanins									
10.	Cyanidin-3-*O*-(6″-*p*-coumaroyl-glucoside)	C_30_H_27_O_13_	2.21	595.14517	−e	595.14572	1.86	287.0544 (100%), 425.0877 (50%)	74911.14	6.40
11.	Pelargonidin-3-*O*-coumarylglucoside	C_30_H_27_O_12_	3.18	579.15025	−e	579.15211	4.15	272.0663 (100%), 563.1574 (20%)	8481.75	0.72
12.	Delphinidin-3-*O*-(6″-*p*-coumaroyl-glucoside)	C_30_H_27_O_14_	0.78	611.14008	−e	611.14093	2.29	303.0505 (100%), 287.0553 (80%)	5366.72	0.46
	Flavonol									
13.	Dihydroquercetin	C_15_H_12_O_7_	3.44	304.05830	+H	305.06674	3.82	163.0395 (100%) 153.0185 (80%)	3683.11	0.31
	Flavans									
14.	Leucocyanidin 4	C_15_H_14_O_7_	2.02	306.07395	+H, +Na	307.08202	2.56	291.0877 (100%), 181.0501 (30%)	52544.44	4.49
15.	Leucopelargonidin	C_15_H_14_O_6_	3.00	290.07904	+H	291.08711	2.75	229.0504 (20%), 165.0551 (100%)	43785.59	3.74
16.	(-)−epigallocatechin	C_15_H_14_O_7_	1.82	306.07395	+H, +Na	307.08240	3.81	263.0532 (100%), 153.0546 (80%)	29619.58	2.53
17.	6-Geranylnaringenin	C_25_H_28_O_5_	4.59	408.19367	+Na	431.18347	1.34	273.0768 (100%), 250.820 (80%)	10237.19	0.87
18.	Naringenin-4′-*O*-glucuronide	C_21_H_20_O_11_	4.08	448.10056	+H	449.10878	2.09	271.0604 (100%), 257.0824 (80%)	10048.21	0.86
19.	Naringenin	C_15_H_12_O_5_	6.21	272.06847	+H	273.07601	0.97	153.0183 (100%)	3875.93	0.33
20.	(+)-Catechin-3-*O*-gallate	C_22_H_18_O_10_	2.21	442.09000	+H	443.09772	1.00	287.0554 (100%), 291.0875 (80%)	3315.07	0.28
	Flavone									
21.	Myricetin-3-*O*-glucoside	C_21_H_20_O_13_	4.48	480.09039	+H, +Na	481.09885	2.46	153.0183 (100%), 319.0456 (90%)	18064.01	1.54
22.	Baicalin hydrate	C_21_H_20_O_12_	3.51	464.09548	+H	465.10369	2.02	285.0766 (100%), 325.0667 (30%)	1838.75	0.16
	Triterpenoid									
23.	Micromeric acid	C_30_H_46_O_3_	11.22	454.34470	+H	455.35284	1.90	383.3285 (100%), 393.3519 (100%), 437.3421 (50%)	194168.06	16.59
24.	Cucurbitacin P	C_30_H_48_O_7_	9.32	520.34000	−e	520.34081	2.60	455.3519 (100%), 337.2736 (80%)	53383.81	4.56
25.	Cucurbitacin F2	C_30_H_46_O_7_	9.70	518.32435	−e	518.32157	−4.32	471.3466 (100%), 355.2645 (30%)	15060.36	1.29
	Amino acid									
26.	Tryptophan	C_11_H_12_N_2_O_2_	1.80	204.08988	+H	205.09785	3.42	188.0714 (100%), 144.0808 (50%)	8815.60	0.75
	*E. hirta* plant extract									
	Phenolic acids									
27.	4-Hydroxycoumarin	C_9_H_6_O_3_	1.76	162.03169	+H	163.03911	0.85	145.0286 (100%)	279416.51	11.14
28.	Caffeoylquinic acid	C_16_H_18_O_9_	2.58	354.09508	+Na, +H	377.08446	0.43	163.0391 (100%), 145.0287 (40%), 177.0548 (30%)	53371.32	2.13
29.	Chlorogenic acid	C_16_H_18_O_9_	1.76	354.09508	+Na, +H	377.08440	0.25	163.0391 (100%), 215.0530 (10%)	40229.57	1.60
30.	3,4-Dihydro-1-benzopyran-2-one	C_9_H_8_O_2_	1.65	148.05243	+H	149.05981	0.73	105.0336 (100%), 123.0441 (30%)	29274.70	1.17
31.	2-S-Glutathionyl caftaric acid	C_23_H_27_N_3_O_15_S	4.58	617.11629	−e	617.11476	−1.59	153.0184 (100%), 307.0608 (60%), 529.1350 (10%), 409.0923 (10%),	25104.15	1.00
32.	Punicalin	C_34_H_22_O_22_	2.42	782.06027	+H	783.06859	1.33	277.0346 (100%), 303.0141 (80%)	19495.48	0.78
33.	4,5-Dicaffeoylquinic acid	C_25_H_24_O_12_	4.67	516.12678	+Na, +H	539.11657	1.07	163.0392 (100%), 499.1239 (50%), 287.0555 (30%)	16282.13	0.65
34.	Feruloyl tartaric acid	C_14_H_14_O_9_	2.25	326.06378	+H	327.07182	2.31	153.0185 (100%), 309.0611 (10%)	13817.19	0.55
35.	Feruloyl malic acid	C_14_H_14_O_8_	0.84	310.06887	−e	310.06978	4.71	200.0449 (100%), 135.0294 (70%)	11092.96	0.44
	Isoflavonoids									
36.	Genistin	C_21_H_20_O_10_	4.81	432.10565	+H, +Na	433.11317	0.56	415.1020 (100%), 397.0920 (80%), 379.0814 (90%)	91534.53	3.65
37.	Dihydrodaidzein-7-*O*-glucuronide	C_21_H_20_O_10_	4.48	432.10565	+H	433.11318	0.59	415.1021 (100%), 255.0646 (80%), 367.0811 (80%)	15379.65	0.61
	Anthocyanins									
38.	Pelargonidin-3-*O*-sambubioside	C_26_H_29_O_14_	4.14	565.15573	−e	565.15593	1.32	547.1449 (100%), 379.0816 (80%)	87443.37	3.49
39.	Pelargonidin-3-*O*-sophoroside	C_27_H_31_O_15_	3.89	595.16630	−e	595.16623	0.81	577.1547 (100%), 271.0596 (90%), 529.1332 (70%), 559.1457 (30%)	58442.79	2.33
40.	Peonidin-3-*O*-arabinoside	C_21_H_21_O_11_	5.58	449.10839	−e	449.10960	3.93	303.0508 (100%), 287.0557 (20%), 413.08617 (10%),	34687.76	1.38
41.	Pelargonidin-3-*O*-coumarylglucoside	C_30_H_27_O_12_	1.67	579.15025	−e	579.15016	0.79	149.0598 (100%), 275.0559 (80%)	15007.82	0.60
	Flavonols									
42.	Quercetin-3-*O*-(6″-malonylglucoside)	C_24_H_22_O_15_	5.24	550.09587	+H, +Na	551.10419	1.90	303.0502 (100%), 345.0609 (20%)	282214.49	11.25
43.	Quercetin-7-*O*-glucoside	C_21_H_20_O_12_	5.02	464.09548	+H, +Na	465.10365	1.93	303.0502 (100%), 433.1132 (30%)	107974.95	4.30
44.	Quercetin-3-*O*-glucuronide	C_21_H_18_O_13_	2.25	478.07474	+H	479.08307	2.19	309.0611 (100%), 303.0521 (805)	58211.34	2.32
45.	Quercetin-3-*O*-rhamnosyl-galactoside	C_27_H_30_O_16_	3.44	610.15338	+H, +Na	611.16197	2.14	153.0184 (100%), 303.0550 (40%)	24565.15	0.98
46.	Quercetin-3-*O*-xylosylglucuronide	C_26_H_26_O_17_	3.85	610.11700	+Na	633.10908	4.52	315.0512 (100%), 319.0457 (90%)	10086.09	0.40
47.	Methylgalangin	C_15_H_10_O_6_	5.81	286.04774	+H	287.05573	2.50	213.0547 (100%), 163.0394 (80%)	9976.99	0.40
	Flavans									
48.	Naringenin-7-*O*-glucuronide	C_21_H_20_O_11_	2.85	448.10056	+H	449.10811	0.60	287.0552 (100%)	206146.03	8.22
49.	Naringenin-4′-*O*-glucuronide	C_21_H_20_O_11_	5.58	448.10056	+Na, +H	471.09085	2.26	303.0508 (100%), 274.0477 (30%), 287.0557 (10%)	59330.32	2.36
	Flavones									
50.	Morin	C_15_H_10_O_7_	5.57	302.04265	+H	303.05090	3.21	153.0187 (100%), 285.0402 (40%),	737073.25	29.38
51.	Kaempferol	C_15_H_10_O_6_	6.09	286.04774	+H	287.05597	3.32	231.0640 (100%), 229.0497 (80%)	68887.11	2.75
52.	Apigenin-7-*O*-apiosyl-glucoside	C_26_H_28_O_14_	4.60	564.14791	+H, +Na	565.15639	2.14	547.1458 (100%), 529.1350 (60%), 303.0503 (40%), 337.0486 (30%)	43427.69	1.73
53.	Luteolin-7-*O*-malonyl-glucoside	C_24_H_22_O_14_	5.82	534.10096	+H, +Na	535.11030	3.86	287.0559 (100%), 163.0394 (40%)	27471.22	1.09
54.	6-Hydroxyluteolin-7-glucoside	C_20_H_18_O_13_	2.86	466.07474	+H	467.08270	1.46	287.0552 (100%), 321.0242 (90%)	9781.46	0.39
55.	Kaempferol-3-*O*-rhamnoside	C_21_H_20_O_10_	6.09	432.10565	+Na, +H	455.09458	−0.64	287.0559 (100%), 153.0186 (60%)	9303.30	0.37
	Triterpenoids									
56.	Cucurbitacin E	C_32_H_44_O_8_	8.37	556.30362	+Na	579.29556	4.71	301.1419 (100%), 277.2167 (50%), 317.2063 (30%)	30532.85	1.22
57.	Cucurbitacin R6	C_30_H_46_O_7_	8.89	518.32435	−e	518.32630	4.82	453.2625 (100%), 184.0736 (80%), 442.2353 (60%), 335.2584 (30%)	9589.35	0.38
	Amino acid									
58.	Tryptophan	C_11_H_12_N_2_O_2_	1.90	204.08988	+H	205.09736	1.00	188.0707 (100%), 170.0601 (90%)	23930.23	0.95
	*A. occidentale* fruits extract									
	Phenolic acid									
59.	Dihydrocaffeic acid-3-*O*-glucuronide	C_15_H_18_O_10_	0.62	358.09000	+Na	381.08111	4.97	198.0526 (100%)	114811.09	33.61
60.	*o*-Coumaric acid	C_9_H_8_O_3_	0.76	164.04734	+H	165.05492	1.81	147.0442 (100%), 109.0657 (70%)	17841.73	5.22
61.	Cinnamoyl glucose	C_15_H_18_O_7_	4.89	310.10525	+Na	333.09472	0.74	204.1017 (100%), 275.0926 (50%)	12415.40	3.63
62.	*p*-Coumaroyl glucose	C_15_H_18_O_8_	2.49	326.10017	+Na	349.08884	−1.57	147.0437 (100%), 119.0492 (70%)	7877.05	2.31
63.	2-Hydroxyphenylacetic acid	C_8_H_8_O_3_	0.80	152.04734	+H	153.05465	0.22	119.0490 (100%), 107.1491 (50%)	6340.16	1.86
64.	4-Hydroxybenzaldehyde	C_7_H_6_O_2_	0.76	122.03678	+H	123.04446	3.25	95.0494 (100%), 107.0491 (70%)	3808.08	1.11
65.	3,4-Dihydro-1-benzopyran-2-one	C_9_H_8_O_2_	4.35	148.05243	+H	149.05992	1.45	131.0494 (100%), 103.0545 (80%)	3467.34	1.01
66.	3,4-Dihydroxyphenylglycol	C_8_H_10_O_4_	0.80	170.05791	+H	171.06566	2.79	139.0388 (100%), 153.0546 (80%)	1729.20	0.51
67.	Salvianolic acid C	C_26_H_20_O_10_	0.73	492.10565	+H	493.11517	4.56	207.0288 (100%), 225.0389 (90%)	1399.04	0.41
	Anthocyanin									
68.	Delphinidin-3-*O*-galactoside	C_21_H_21_O_12_	4.89	465.10330	−e	465.10344	1.49	303.0502 (100%)	2014.41	0.59
	Flavonols									
69.	Quercetin-3-*O*-galactoside	C_21_H_20_O_12_	4.88	464.09548	+Na, +H	487.08535	1.34	153.0184 (100%), 303.0502 (20%)	8556.56	2.50
70.	Dihydroquercetin-3-*O*-rhamnoside	C_21_H_22_O_11_	3.00	450.11621	+Na	473.10555	0.25	303.0517 (100%)	1800.84	0.53
	Flavans									
71.	3′-*O*-Methyl-(-)−epicatechin-7-*O*-glucuronide	C_22_H_24_O_12_	4.35	480.12678	+H	481.13465	1.23	313.0710 (100%), 245.0470 (20%)	8248.27	2.41
72.	Naringenin-5-*O*-glucuronide	C_21_H_20_O_11_	5.49	448.10056	+Na, +H	471.09023	0.96	303.0501 (100%), 287.0552 (30%)	2980.31	0.87
73.	(-)−epigallocatechin	C_15_H_14_O_7_	0.82	306.07395	+H	307.08107	−0.52	263.0532 (100%), 153.0546 (80%)	2464.54	0.72
74.	6-Prenylnaringenin	C_20_H_20_O_5_	4.00	340.13107	+H	341.13985	4.39	323.1282 (100%), 193.0859 (80%)	2278.05	0.67
75.	Leucopelargonidin-3-*O*-alpha-l-rhamno-beta-d-glucopyranoside	C_27_H_34_O_15_	4.67	598.18977	+H, +Na	599.19741	0.60	495.1482 (100%), 374.1589 (30%), 290.0399 (20%)	2086.70	0.61
	Flavones									
76.	Myricetin	C_15_H_10_O_8_	4.67	318.03757	+H	319.04544	1.88	153.0182 (100%), 165.0183 (30%)	9795.79	2.87
77.	Morin	C_15_H_10_O_7_	5.49	302.04265	+H	303.05049	1.86	287.0552 (90%), 153.0437 (100%)	8258.29	2.42
78.	Isovitexin	C_21_H_20_O_10_	4.71	432.10565	+H, +Na	433.11372	1.85	337.0715 (100%), 415.1022 (90%), 283.0605 (80%)	5879.40	1.72
79.	Myricetin-3-*O*-glucoside	C_21_H_20_O_13_	4.23	480.09039	+Na, +H	503.08111	2.97	319.0453 (100%)	4580.01	1.34
	Flavanone									
80.	Pinocembrin	C_15_H_12_O_4_	6.09	256.07356	+H	257.08090	0.24	153.0185 (100%)	2037.62	0.60
	Lactone									
81.	Coumarin	C_9_H_6_O_2_	0.76	146.03678	+H	147.04406	0.04	123.0442 (100%), 95.0494 (50%)	3826.69	1.12
	Chalcon									
82.	Phloretin	C_15_H_14_O_5_	4.59	274.08412	+H	275.09162	0.79	131.0491 (100%), 151.0390 (70%), 133.0649 (60%)	3254.60	0.95
	Triterpenoids									
83.	Micromeric acid	C_30_H_46_O_3_	8.55	454.34470	+H	455.35167	-0.66	437.3409 (100%), 423.3296 (20%)	54817.17	16.05
84.	Cucurbitacin E	C_32_H_44_O_8_	8.37	556.30362	+Na	579.29310	0.45	301.1407 (100%)	24481.80	7.17
85.	Cucurbitacin F2	C_30_H_46_O_7_	9.72	518.32435	−e	518.32215	−3.19	471.3475 (100%), 454.2935 (50%)	11253.75	3.29
86.	Cucurbitacin R6	C_30_H_46_O_7_	8.72	518.32435	−e	518.32544	3.16	335.2584 (100%), 184.0736 (80%), 361.2357 (40%)	6412.54	1.88
87.	Cucurbitacin P	C_30_H_48_O_7_	9.12	520.34000	−e	520.34005	1.14	337.2739 (100%), 398.2676 (30%)	5347.24	1.57
	Fatty acid									
88.	3-Hydroxyphenylvaleric acid	C_11_H_14_O_3_	7.66	194.09429	+H	195.10218	3.10	95.0493 (100%)	1556.22	0.46

## References

[1] Lim W.Y., Wong C.W. (2018). Inhibitory effect of chemical and natural anti-browning agents on polyphenol oxidase from ginger (Zingiber officinale Roscoe). J. Food Sci. Technol..

[2] Loizzo M.R., Tundis R., Menichini F. (2012). Natural and Synthetic Tyrosinase Inhibitors as Antibrowning Agents: An Update. Compr. Rev. Food Sci. Food Saf..

[3] Lim W.Y., Cheun C.F., Wong C.W. (2019). Inhibition of enzymatic browning in sweet potato (Ipomoea batatas (L.)) with chemical and natural anti-browning agents. J. Food Process. Preserv..

[4] Paudel P., Seong S.H., Wagle A., Min B.S., Jung H.A., Choi J.S. (2020). Antioxidant and anti-browning property of 2-arylbenzofuran derivatives from Morus alba Linn root bark. Food Chem..

[5] Botterweck A.A.M., Verhagen H., Goldbohm R.A., Kleinjans J., Van Den Brandt P.A. (2000). Intake of butylated hydroxyanisole and butylated hydroxytoluene and stomach cancer risk: Results from analyses in the Netherlands Cohort Study. Food Chem. Toxicol..

[6] Zolghadri S., Bahrami A., Tareq M., Khan H., Munoz-munoz J., Garcia-molina F. (2019). A comprehensive review on tyrosinase inhibitors. J. Enzyme Inhib. Med. Chem..

[7] Afaq F., Katiyar S.K. (2011). Polyphenols: Skin Photoprotection and Inhibition of Photocarcinogenesis. Mini. Rev. Med. Chem..

[8] Chang T. (2009). An Updated Review of Tyrosinase Inhibitors. Int. J. Mol. Sci..

[9] Lyu X., Lee J., Chen W.N. (2019). Potential natural food preservatives and their sustainable production in yeast: Terpenoids and polyphenols. J. Agric. Food Chem..

[10] Carocho M., Barreiro M.F., Morales P., Ferreira I.C.F.R. (2014). Adding molecules to food, pros and cons: A review on synthetic and natural food additives. Compr. Rev. Food Sci. Food Saf..

[11] Faustino M., Veiga M., Sousa P., Costa E.M., Silva S., Pintado M. (2019). Agro-food byproducts as a new source of natural food additives. Molecules.

[12] Boutanaev A.M., Moses T., Zi J., Nelson D.R., Mugford S.T., Peters R.J., Osbourn A. (2014). Investigation of terpene diversification across multiple sequenced plant genomes. Proc. Natl. Acad. Sci. USA.

[13] Chang T.S. (2012). Natural melanogenesis inhibitors acting through the down-regulation of tyrosinase activity. Materials.

[14] Rouf A., Naik H.R., Beigh M.A., Kanojia V., Mir S.A., Aafia S., Ayaz Q., Altaf U. (2018). Enzymatic browning of apple and its control by chemical treatment: A review. Int. J. Food Sci. Nutr..

[15] Jdey A., Falleh H., Ben Jannet S., Mkadmini Hammi K., Dauvergne X., Ksouri R., Magné C. (2017). Phytochemical investigation and antioxidant, antibacterial and anti-tyrosinase performances of six medicinal halophytes. South African J. Bot..

[16] Haliloglu Y., Ozek T., Tekin M., Goger F., Baser K.H.C., Ozek G. (2017). Phytochemicals, antioxidant, and antityrosinase activities of Achillea sivasica Çelik and Akpulat. Int. J. Food Prop..

[17] Welch C., Zhen J., Bassène E., Raskin I., Simon J.E., Wu Q. (2018). Bioactive polyphenols in kinkéliba tea (Combretum micranthum) and their glucose-lowering activities. J. Food Drug Anal..

[18] De Morais Lima G.R., De Sales I.R.P., Filho M.R.D.C., De Jesus N.Z.T., De Sousa Falcão H., Barbosa-Filho J.M., Cabral A.G.S., Souto A.L., Tavares J.F., Batista L.M. (2012). Bioactivities of the genus Combretum (Combretaceae): A review. Molecules.

[19] Kausar J., Muthumani D., Hedina A., Anand V. (2016). Review of the phytochemical and pharmacological activities of Euphorbia hirta Linn. Pharmacogn. J..

[20] Al-Snafi A.E. (2017). Pharmacology and therapeutic potential of Euphorbia hirta (Syn: Euphorbia pilulifera)—A review. J. Pharm..

[21] Dedehou E., Dossou J., Anihouvi V., Soumanou M.M. (2016). A review of cashew (Anacardiumoccidentale L.) apple: Effects of processing techniques, properties and quality of juice. African J. Biotechnol..

[22] Cunha A.G., Brito E.S., Moura C.F.H., Ribeiro P.R.V., Miranda M.R.A. (2017). UPLC–qTOF-MS/MS-based phenolic profile and their biosynthetic enzyme activity used to discriminate between cashew apple (Anacardium occidentale L.) maturation stages. J. Chromatogr. B Anal. Technol. Biomed. Life Sci..

[23] Yi W., Wei Q., Di G., Jing-yu L., Yang-li L. (2012). Phenols and flavonoids from the aerial part of Euphorbia hirta. Chin. J. Nat. Med..

[24] Radojkovi M., Zekovi Z., Joki S., Vidovi S., Milo S. (2012). Optimization of Solid-Liquid Extraction of Antioxidants from Black Mulberry Leaves by Response Surface Methodology. Food Technol. Biotechnol..

[25] Liu Y., Zheng Y., Wang A. (2011). Response Surface Methodology for Optimizing Adsorption Process Parameters for Methylene Blue Removal by a Hydrogel Composite. J. Adsorpt. Sci. Technol..

[26] Bezerra M.A., Santelli R.E., Oliveira E.P., Villar L.S., Escaleira L.A. (2008). Response surface methodology (RSM) as a tool for optimization in analytical chemistry. Talanta.

[27] Taha F.S., Mohamed G.F., Mohamed S.H., Mohamed S.S., Kamil M.M. (2011). Optimisation of the Extraction of Total Phenolic Compounds from Sunflower Meal and Evaluation of the Bioactivities of Chosen Extracts. Am. J. Food Technol..

[28] Ghafoor K., Choi Y.H., Jeon J.Y., Jo I.H. (2014). Optimization of Ultrasound-Assisted Extraction of Phenolic Compounds, Antioxidants, and Anthocyanins from Grape (Vitis vinifera) Seeds. J. Agric. Food Chem..

[29] Xu D., Zhou Y., Zheng J., Li S., Li A., Li H. (2016). Optimization of Ultrasound-Assisted Extraction of Natural Antioxidants from the Flower of Jatropha integerrima by Response Surface Methodology. Molecules.

[30] Amirah D.M., Khan M.R. (2012). Comparison of Extraction Techniques on Extraction of Gallic Acid from Stem Bark of Jatropha curcas. J. Appl. Sci..

[31] Altemimi A., Watson D.G., Choudhary R., Dasari M.R. (2016). Ultrasound Assisted Extraction of Phenolic Compounds from Peaches and Pumpkins. PLoS ONE.

[32] Liao J., Qu B., Zheng N. (2016). Effects of Process Parameters on the Extraction of Quercetin and Rutin from the Stalks of Euonymus Alatus (Thumb.) Sieb and Predictive Model Based on Least Squares Support Vector Machine Optimized by an Improved Fruit Fly Optimization Algorithm. Appl. Sci..

[33] Charpe T.W., Rathod V.K. (2014). Effect of Ethanol Concentration in Ultrasound Assisted Extraction of Glycyrrhizic Acid from Licorice Root. Iran. J. Chem. Eng..

[34] Gutte K.B., Sahoo A.K., Ranveer R.C. (2015). Effect of ultrasonic treatment on extraction and fatty acid profile of flaxseed oil. OCL.

[35] Alzeer J., Abou Hadeed K. (2016). Ethanol and its Halal status in food industries. Trends Food Sci. Technol..

[36] Pintus F., Span D., Corona A., Medda R. (2015). Antityrosinase activity of Euphorbia characias extracts. PeerJ.

[37] Yuet Ping K., Darah I., Chen Y., Sreeramanan S., Sasidharan S. (2013). Acute and subchronic toxicity study of euphorbia hirta L. methanol extract in rats. Biomed Res. Int..

[38] Rajeh M.A.B., Kwan Y.P., Zakaria Z., Latha L.Y., Jothy S.L., Sasidharan S. (2012). Acute toxicity impacts of Euphorbia hirta L extract on behavior, organs body weight index and histopathology of organs of the mice and Artemia salina. Pharmacogn. Res..

[39] Innocent B.Y., Daniel A., Mikponsè D.A.A., Frédéric L., Raphaël D., Evelyne L., Célestin T.C.K., Sachi P., Roseline B., Apollinaire M.G. (2016). Histopathological study of toxicity mixture of cashew apple juice (Anacardium occidentale), cow milk and yogurt on the wistar rat. J. Exp. Biol. Agric. Sci..

[40] Kpemissi M., Metowogo K., Melila M., Veerapur V.P., Negru M., Taulescu M., Potârniche A.V., Suhas D.S., Puneeth T.A., Vijayakumar S. (2020). Acute and subchronic oral toxicity assessments of Combretum micranthum (Combretaceae) in Wistar rats. Toxicol. Rep..

[41] Pióro-Jabrucka E., Pawełczak A., Przybył J.L., Węglarz Z. (2011). Accumulation of phenolic and sterol compounds in Euphorbia hirta (L.). Herba Pol..

[42] Suênia M., Vilar D.A., De Souza G.L., Vilar D.D.A., Leite J.A., Raffin F.N., Barbosa-Filho J.M., Henrique F., Nogueira A., Rodrigues-Mascarenhas S. (2016). Assessment of Phenolic Compounds and Anti-Inflammatory Activity of Ethyl Acetate Phase of Anacardium occidentale L. Bark. Molecules.

[43] Sampaio B.L., Edrada-Ebel R., Batista F., Costa D. (2016). Effect of the environment on the secondary metabolic profile of Tithonia diversifolia: A model for environmental metabolomics of plants. Sci. Rep..

[44] Yang Z., Zhang Y., Sun L., Wang Y., Gao X., Cheng Y. (2012). Analytica Chimica Acta An ultrafiltration high-performance liquid chromatography coupled with diode array detector and mass spectrometry approach for screening and characterising tyrosinase inhibitors from mulberry leaves. Anal. Chim. Acta.

[45] Wang B., Juang L., Yang J., Chen L., Tai H., Huang M. (2012). Antioxidant and Antityrosinase Activity of Flemingia macrophylla and Glycine tomentella Roots. Evid.-Based Complement. Altern. Med..

[46] Wang Y., Zhang G., Yan J., Gong D. (2014). Inhibitory effect of morin on tyrosinase: Insights from spectroscopic and molecular docking studies. Food Chem..

[47] Asthana S., Zucca P., Vargiu A.V., Sanjust E., Ruggerone P., Rescigno A. (2015). Structure—Activity Relationship Study of Hydroxycoumarins and Mushroom Tyrosinase. J. Agric. Food Chem..

[48] Taofiq O., González-Paramás A.M., Barreiro M.F., Ferreira I.C.F.R., McPhee D.J. (2017). Hydroxycinnamic acids and their derivatives: Cosmeceutical significance, challenges and future perspectives, a review. Molecules.

[49] Vardanega R., Santos D.T., Meireles M.A.A. (2014). Intensification of bioactive compounds extraction from medicinal plants using ultrasonic irradiation. Pharmacogn. Rev..

[50] Li H., Zhang Z., Xue J., Cui L., Hou T., Li X., Chen T. (2016). Optimization of ultrasound-assisted extraction of phenolic compounds, antioxidants and rosmarinic acid from perilla leaves using response surface methodology. Food Sci. Technol..

[51] Zhen-Ming L., Zhe G.J., Hong-yu X., Wen-fang D., Zheng-hong S.J. (2011). Optimization of Extraction of Total Triterpenoids from Submergedly Cultured Antrodia camphorata using Response Surface Methodology. Nat. Prod. Res. Dev..

[52] El Khoury R., Michael R., Marc J., Beyrouthy E., Baillet A., Toufic G., Ali R., Roger T. (2018). Phytochemical screening and antityrosinase activity of carvacrol, thymoquinone, and four essential oils of Lebanese plants. J. Cosmet Dermatol..

[53] Kumar T., Khan Z., Oulkar D., Singh B.K., Maurya A., Singh B., Banerjee K. (2017). High resolution LC-MS characterization of phenolic compounds and the evaluation of antioxidant properties of a tropical purple radish genotype. Arab. J. Chem..

[54] Sahin S., Aybastıer Ö., Isik E. (2013). Optimisation of ultrasonic-assisted extraction of antioxidant compounds from Artemisia absinthium using response surface methodology. Food Chem..

[55] Amado I.R., Franco D., Sanchez M., Zapata C., Vazquez J.A. (2014). Optimisation of antioxidant extraction from Solanum tuberosum potato peel waste by surface response methodology. Food Chem..

